# Role of Antioxidants in Neonatal Hypoxic–Ischemic Brain Injury: New Therapeutic Approaches

**DOI:** 10.3390/ijms18020265

**Published:** 2017-01-28

**Authors:** Olatz Arteaga, Antonia Álvarez, Miren Revuelta, Francisco Santaolalla, Andoni Urtasun, Enrique Hilario

**Affiliations:** 1 Department of Cell Biology & Histology, School of Medicine and Nursing, University of the Basque Country (UPV/EHU), 48940 Leioa, Spain; antoniaangeles.alvarez@ehu.eus (A.Á.); mrevuelta006@gmail.com (M.R.); enrique.hilario@ehu.eus (E.H.); 2 Department of Otorhinolaryngology, Basurto University Hospital, School of Medicine and Nursing, University of the Basque Country (UPV/EHU), 48940 Leioa, Spain; francisco.santaolalla@ehu.eus; 3 Department of Neuroscience, School of Medicine and Nursing, University of the Basque Country (UPV/EHU), 48940 Leioa, Spain; andoni.urtasun@ehu.eus; 4 Neurogenomiks Laboratory, Achucarro Basque Center for Neuroscience, Bizkaia Science and Technology Park, 48170 Zamudio, Spain

**Keywords:** antioxidant, neuroprotection, hypoxia–ischemia, brain, newborn

## Abstract

Hypoxic–ischemic brain damage is an alarming health and economic problem in spite of the advances in neonatal care. It can cause mortality or detrimental neurological disorders such as cerebral palsy, motor impairment and cognitive deficits in neonates. When hypoxia–ischemia occurs, a multi-faceted cascade of events starts out, which can eventually cause cell death. Lower levels of oxygen due to reduced blood supply increase the production of reactive oxygen species, which leads to oxidative stress, a higher concentration of free cytosolic calcium and impaired mitochondrial function, triggering the activation of apoptotic pathways, DNA fragmentation and cell death. The high incidence of this type of lesion in newborns can be partly attributed to the fact that the developing brain is particularly vulnerable to oxidative stress. Since antioxidants can safely interact with free radicals and terminate that chain reaction before vital molecules are damaged, exogenous antioxidant therapy may have the potential to diminish cellular damage caused by hypoxia–ischemia. In this review, we focus on the neuroprotective effects of antioxidant treatments against perinatal hypoxic–ischemic brain injury, in the light of the most recent advances.

## 1. Introduction

Perinatal hypoxic–ischemic encephalopathy (HIE) still continues to be an alarming health and economic problem, being associated with approximately 25% of global neonatal deaths [1,2]. This condition affects 1.5 per 1000 newborns annually [3]. However, the incidence is even higher in low- and middle-income countries, where 96% of the 1.15 million cases of neonatal encephalopathy estimated in 2010 took place [1,4]. In fact, intrapartum hypoxia–ischemia (HI) is associated with an estimated 30% of the cases of neonatal encephalopathy in developed countries and 60% of cases in developing countries [3].

Thus, HIE is believed to be one of the most important causes of mortality and chronic disorders in infants [5,6]. The different causes of HIE can happen before, during or after birth. Premature neonates are particularly at risk of suffering HIE, with the incidence being a significant 60% higher [7,8]. Due to the reduction of oxygen and glucose in the brain, newborns can die or suffer from severe neurological dysfunctions such as cerebral palsy, mental retardation, epilepsy, visual and hearing impairment, learning and behavioral disabilities, attention deficits and hyperactivity [9,10,11,12].

**Figure 1:**
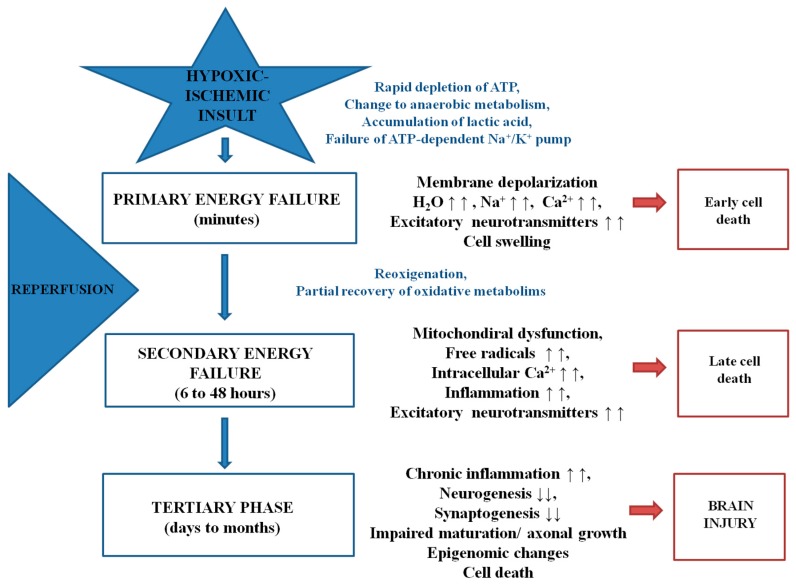
Pathogenic mechanisms involved in neonatal hypoxia–ischemia. Primary energy failure occurs immediately after the hypoxic–ischemic insult. After reperfusion, there is a secondary energy failure, which can extend in duration from 6 to 48 h. Finally, the tertiary phase takes place and the previous deleterious events can further exacerbate brain injury (from days to months). The up arrows represent an increase while the down arrows show a decrease on the corresponding metabolite/process.

The pathogenic mechanisms of HI injury have recently been classified into three phases: that of primary energy failure due to the HI insult; a secondary phase, which is a consequence of reoxygenation and reperfusion and, finally, a tertiary phase in which previous events can get worse and inflammation become chronic [5,13,14,15] (Figure 1). Briefly, when the hypoxic–ischemic insult occurs, there is a rapid depletion of ATP due to the decrease in oxidative phosphorylation. Although the cell changes to anaerobic metabolism, this is energetically inefficient and results in the failure of the ATP-dependent Na/K pump and accumulation of metabolites, such as lactic acid and hypoxanthine. Then, a progressive membrane depolarization occurs, with an excessive accumulation of excitatory amino acids on the extracellular side, as well as an excessive entry of water, sodium and calcium into the cell. All these primary processes lead to cellular edema and early cell death. The second phase is related to reoxygenation and reperfusion. The partial recovery of oxidative metabolism triggers an increased production of radical oxygen species (ROS), higher levels of intracellular calcium and mitochondrial dysfunction. There is also an increase in the expression of pro-inflammatory genes and late cell death. Finally, previously mentioned harmful processes can be exacerbated in the tertiary phase, which can last from days to months. While neurogenesis, synaptogenesis and axonal growth are diminished, inflammation and epigenomic changes augment, leading to detrimental brain injury.

## 2. Oxidative Stress and Endogenous Antioxidants

Perinatal hypoxic–ischemic injury causes devastating brain damage and there is little doubt about the fact that oxidative stress is one of the mayor contributors to this. An excessive production of ROS occurs, including radical species, such as superoxide and hydroxyl radicals, as well as non-radical toxic species, such as singlet oxygen and hydrogen peroxide [16]. Not only are ROS overproduced, but also reactive nitrogen species, with nitric oxide being the most detrimental, since this can significantly enhance the toxicity of superoxide radicals [13].

**Figure 2:**
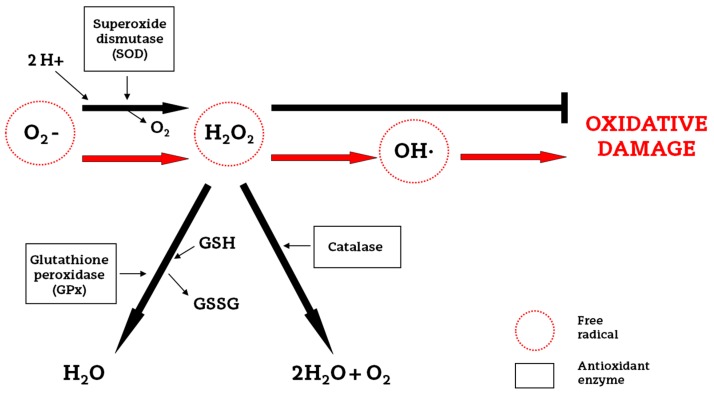
Effects of antioxidant enzymes on free radicals in the neonatal brain. In the diagram, the cell’s enzymatic antioxidant defense system, consisting of three enzymes is shown. Superoxide dismutase (SOD) catalyzes the dismutation of the superoxide radical (O_2_^−^) to hydrogen peroxide (H_2_O_2_). Then, glutathione peroxidase (GPx) together with catalase catalyze the reduction of H_2_O_2_ to water and oxygen. In the absence of GPx and catalase, the O_2_^−^ is converted to hydroxyl radical (OH^−^), which can induce oxidative damage to the cell. GSH, glutathione; GSSG, glutathione disulfide. The black arrows represent the mitigation or avoidance of the oxidative damage due to the antioxidant enzymes, while the red arrows represent the free radical production in absence of an efficient antioxidant defense system which leads to an oxidative damage process.

The brain, apart from being the most metabolically active organ in the human body, maintains a fragile redox homeostasis, because of its low antioxidant defense and high content of easily oxidizable membrane lipids. Despite the fact that under physiological conditions the antioxidant enzyme system is able to protect against cell injury, under asphyctic conditions it fails, with neurons being particularly vulnerable to free radical damage [17]. The cellular enzymatic antioxidant defense system consists of three enzymes: superoxide dismutase (SOD), which catalyzes the dismutation of the superoxide radical to hydrogen peroxide (H_2_O_2_); glutathione peroxidase (GPx) and catalase, which catalyze the reduction of H_2_O_2_ to water and oxygen [13] (Figure 2).

The infant brain is particularly susceptible to HI [18,19], because of its specific characteristics: abundant unsaturated fatty acids, high rate of oxygen consumption, few antioxidants, high water content, low myelinization, low concentration of antioxidant enzymes, higher concentrations of free iron and oxygen-induced vasoconstriction [20,21,22,23,24]. The implication of ROS in the HI-induced brain damage is irrefutable; for example, it has been clearly shown that plasma malondialdehyde, a product of lipid peroxidation, and nitrate/nitrite levels were significantly higher in neonates with HIE [25]. During reperfusion and reoxygenation, the antioxidant defense system is overwhelmed by the oxidative stress, leading to further damage: lipid peroxidation, protein denaturation, inactivation of enzymes, DNA damage, release of calcium from intracellular stores and damage to the cytoskeletal structure [26]. Hence, cell membrane are highly susceptible to free radical-induced peroxidation because of their polyunsaturated fatty acids [25] particularly in the immature brain.

## 3. New Approaches to Antioxidant Therapy

Nowadays, therapeutic hypothermia has been established as a standard clinical procedure for infants diagnosed with moderate to severe neonatal encephalopathy in the developed world [27], supported by evidence from both animal studies and large-scale clinical trials in humans [28]. Numerous studies performed on animals have demonstrated that hypothermia is able to ameliorate long-term brain injury in both the grey and white matter in rats [29] and to reduce brain cell death in a piglet asphyxia model with cooling by 3.5 to 5 °C [30]. However, this study also reported that following cooling by 8.5 °C cell death was increased in piglets, suggesting the potential detrimental effects of excessive cooling and underlining the importance of research about the optimal cooling temperature.

Concerning the effects of hypothermia in humans, Jacobs et al. [31] reviewed 11 randomized controlled human trials involving 1505 term and late preterm neonates with moderate or severe encephalopathy and evidence of intrapartum asphyxia and concluded that cooling significantly reduced the mortality rate, with the benefits outweighing the short-term adverse effects. Nevertheless, therapeutic hypothermia is only partially effective, with almost 50% of treated infants having adverse outcomes [32]. More specifically, in the United Kingdom, 45% of neonates have an adverse outcome after HI despite hypothermia, with 25% dying and 20% developing cerebral palsy and other life-long debilitating conditions [32]. Adjunct therapies for hypothermia are needed to enhance overall protection and improve outcomes. Moreover, in low- and mid-resource settings where cooling is not routine, alternative therapies may be important [33].

Various studies have shown that the efficacy associated with therapeutic hypothermia could be increased by using a combination of treatments [27]. For example, Park et al. [34] found that hypothermia augments the neuroprotective activity of mesenchymal stem cells derived from human umbilical cord blood for neonatal HIE in male rats [34]. In addition, Lafuente et al. [35] reported the additive protective effects of hypothermia and cannabidiol on excitotoxicity, inflammation and oxidative stress, and on cell damage; the effects of combination therapy were greater than either hypothermia or cannabidiol alone. Although multiple therapeutic strategies for the amelioration of neonatal HIE have been investigated at the experimental level [5,36], the corresponding results have not been as positive as expected. Since increased levels of oxidative stress, excitatory neurotransmitters and inflammation occurring during the HI insult are considered to be the “deadly triad” leading to brain damage [37,38], it is reasonable to assume that therapies aimed at preventing the damage caused by all these processes may afford effective and even complete neuroprotection [37,38,39].

The developing neonatal brain is particularly vulnerable to oxidative stress because free-radical scavenging systems have not yet matured, resulting in insufficient synthesis of antioxidant enzymes/scavengers following injury [33]. As antioxidants can safely interact with free radicals and terminate that chain reaction before vital molecules are damaged, it seems reasonable to assume that exogenous antioxidant therapy may have the potential to reduce the associated cellular damage. In fact, antioxidants might act at different steps of the damage: scavenging ROS, reducing the production of free radicals, altering antiradical defenses, increasing the antioxidant levels and adding lipophilic antioxidants into cell membranes [40]. For this reason, we review here the most recent advances regarding antioxidant therapies for perinatal hypoxic–ischemic brain injury.

### 3.1. Allopurinol

Allopurinol is a chemical compound which is able to inhibit xanthine oxidase and acts as a free radical scavenger. Free radical formation due to conversion of hypoxanthine into xanthine by xanthine oxidase is very important during HI injury. In fact, during the first phase of HI when cells shift to anaerobic metabolism, hypoxanthine accumulates, and during the reoxygenation phase, hypoxanthine is oxidized by xanthine oxidase and superoxide free radicals are produced [41]. This is why the inhibition of xanthine oxidase by allopurinol is fundamental to avoid the propagation of ROS production during the first phases of HI insult. Although allopurinol has scarcely been investigated in animal models of HI brain injury, it has been shown to exhibit neuroprotective properties when administered 15 min after inducing hypoxia–ischemia in 7-day-old (P7) Wistar rats at a high dose (Zyloprim sodium, 135 mg/kg). It ameliorated brain injury by reducing the increase in the water content in the ipislateral hemisphere and markedly decreasing atrophy, and it also reduced long-term cerebral injury in immature rats [42,43]. More recently, the effects of allopurinol administration prior to hypoxia on brain levels of adenosine and purine metabolites have been evaluated in a larger animal model [44]. In the newborn piglet, allopurinol effectively inhibited xanthine oxidase and resulted in a significant increase in brain tissue levels of adenosine and inosine under severe hypoxic conditions.

In spite of the paucity of research performed on animals, the number of human clinical trials evaluating the efficacy of allopurinol against HIE is higher. However, the results obtained from these studies are contradictory. On the one hand, some studies were not found to be as promising as expected. Benders et al. [45] claimed that early postnatal allopurinol (Apurin, 40 mg/kg), administered intravenously as soon as possible after birth asphyxia (about 4 h after birth) and 12 h later, does not reduce the early reperfusion-induced free radical surge and does not improve short term outcome after severe birth asphyxia, although the authors also pointed out that their sample size was too small to exclude beneficial effects of early postnatal allopurinol treatment. Besides, Chaudhari and McGuire [46] reviewed the available data about randomized or quasi-randomized controlled trials that compared allopurinol vs. placebo in newborns with HIE, and they concluded that the available data could not conclusively demonstrate the benefits of this compound, as much larger trials were needed. On the other hand, other clinical trials have successfully demonstrated the neuroprotective effect of this xanthine oxidase inhibitor in neonatal HIE. A high dose of allopurinol (40 mg/kg) has been found to have beneficial effects on free radical formation, cerebral blood volume and electrical brain activity, without toxic side effects [47]. Moreover, it has been proved that allopurinol (500 mg), intravenously (i.v.) administered to the mother during fetal hypoxia crosses the placenta and lowers cord blood levels of the brain injury marker S-100B, as well as nonprotein-bound iron concentrations [48]. Similarly, in another trial, a group of grade I to III (mild to severe) asphyxiated infants were treated with intravenous allopurinol (Apuria; 40 mg/kg/day) for 3 days (with the first dose within 2 h after birth; total dose of 40 mg/kg administered in 30 min in two dosages for 3 days). Reduced serum nitric oxide levels were observed, but high nitric oxide levels were found to be present in severely asphyxiated infants. Additionally, the asphyxiated newborns treated with allopurinol had better neurologic and neurodevelopmental outcomes at 12 or more months of age [49]. More recently, Kaandorp et al. [50] found that neonatal allopurinol (Apurin) treatment may lower the risk of death or severe disability in the long term (at 4–8 years of age) in moderately asphyxiated infants, indicating long-term neuroprotective effects, and that neonatal treatment with high-dose allopurinol does not have negative side effects in the follow-up of two randomized controlled trials, where infants received 40 mg/kg allopurinol (with an interval of 12 h) starting within 4 h after birth. Therefore, the majority of the pilot studies indicate that allopurinol can exert long-term neuroprotection in human infants, by reducing free-radical production and by acting as a free-radical scavenger and free iron chelator, without having negative side effects when administered at high and repeated doses.

### 3.2. Erythropoietin

Erythropoietin (EPO) is a glycoprotein hormone that controls erythropoiesis and is also a pleiotropic cytokine with a number of erythropoietic and non-erythropoietic roles [51]. EPO and EPO-receptor (EPO-R) signaling are required for normal brain development [52]; in fact, during gestation, there is an over-expression of both, and after birth their expression is diminished quickly in human and rodent brains. After hypoxia, the expression of hypoxia inducible factor-1 (HIF-1) is stable, and there is an increase in the expression of downstream targets and growth factors such as EPO and vascular endothelial growth factor [53,54]. Consequently, EPO and EPO-R are specifically expressed by neurons, astrocytes and microglia [55].

EPO has been shown to be effective in adult rodent models of transient focal ischemia [56] and permanent ischemia [55] by reducing infarct volume, and in embolic stroke by enhancing neurogenesis and angiogenesis and improving neurological function [57]. Moreover, varying evidence points to the efficacy of EPO against HIE in rodent and primate models, since this hormone exhibits anti-inflammatory, anti-apoptotic and antioxidant effects [58,59], as detailed below. Nevertheless, the cellular mechanisms by which EPO exerts neuroprotection are not completely understood [51].

In the Vannucci model, the neuroprotective effect of erythropoietin against brain damage has been demonstrated under many different conditions: given as pre-treatment or as post-treatment and with a single or multiple dose administration. However, not all the studies were carried out under the same conditions, which hinders the extraction of clear conclusions. In the case of recombinant human EPO (rhEPO) (300 units) administered intraperitoneally (i.p.) 24 h before hypoxia but repeated (300 units) daily for an additional 2 days, reduced apoptotic cell death, partially mediated by the activation of heat shock protein 27 in unsexed P7 Sprague Dawley rats, was reported [58]. When a high dose rhEPO (1000 U/kg) was given intraperitoneally to 6-day-old Wistar rats immediately after a HI insult, significantly decreased mean infarct volume [60], and significantly improved long-term neurobehavioral achievements, as well as significantly diminished brain injury, in particular spared hippocampal CA1 neurons, were found [61]. However, Fan et al. [62] reported that rhEPO (Eprex; i.p., 5 or 20 kU/kg at 0, 24, and 48 h after HI) was not neuroprotective at a histological level in a HI model using P9 mice (C57B1/6 J), when determined 24 h after the last EPO treatment (72 h after HI). In contrast, long-term improvement in sensorimotor function, striatum atrophy, hippocampal lesions, and white matter injury were observed when using 5 kU/kg EPO, but curiously only in female animals. These authors suggested that EPO presents an inverted U-shaped dose–response curve, since the beneficial effects of the lower EPO concentration were stronger and lasted longer than those of a higher dose. These results were in accordance with those of Kellert et al. [63] who reported that three doses of 5000 U/kg subcutaneous rhEPO beginning immediately after the injury is optimal because it limits total drug exposure and provides maximal benefit.

Concerning the time of delivery, Alexander et al. [64] performed a study designed to assess the effectiveness of immediate and delayed administration of EPO on long-term behavioral and histological outcomes in P7 male Wistar rats with early HI injury. Results from that study showed a therapeutic benefit of EPO when given immediately following induction of HI injury, with diminished benefits from a 60 min delayed injection of EPO, and no protection following a 180 minute delayed injection. However, they used a single intraperitoneal injection schedule (1000 U/kg), whereas according to the majority of articles, a repeated dosage of EPO is required for protection to become evident.

In other rodent models of hypoxic–ischemic brain injury, rhEPO has also demonstrated its neuroprotective properties. Delayed erythropoietin therapy (1000 U/kg per dose × three doses) improves histological and behavioral outcomes at one month following transient neonatal stroke induced by transient middle cerebral artery occlusion for 3 h in P10 Sprague-Dawley rats [65]. These results suggest that delayed EPO therapy may provide a late treatment alternative for early brain injury. Similarly, in a model of transient middle cerebral artery occlusion, multiple doses of rhEPO (R&D Systems; 1000 U/kg, at reperfusion, 24 h, and 7 days later) increased the neurogenesis and oligodendrogliosis of subventricular zone precursor cells in P7 Long Evans rats [66]; it also sustains cognitive function and brain volume after neonatal stroke in P10 Sprague-Dawley rats [67]. In this model as well, immediately upon reperfusion, a single dose of rhEPO (5 units per gram of body weight) preserved brain tissue and decreased infarct volume 6 weeks after injury; it also increased the percentage of newly born neurons in the injured areas. Both pre-treatment (one hour before) and post-treatment (immediately after insult, followed by additional injections at 24 and 48 h post-insult) with rhEPO (5 U/g) ameliorates neonatal HI-induced neurobehavioral deficits, neuroinflammation, and hippocampal injury in the juvenile rat model of HI (P5), which is relevant to immature human infants [68]. rhEPO also protected against white matter damage in a preterm equivalent neonatal HI model in two-day-old rats [69]. Furthermore, postnatal recombinant erythropoietin (2000 U/kg/dose, once a day from postnatal P1 to P5) was found to mitigate impaired cerebral cortical development following subplate loss [70] and promote oligodendrocyte development [71] in a prenatal transient systemic HI model performed on embryonic day 18.

The effectiveness of EPO in combination with hypothermia has been demonstrated in a term nonhuman primate model of perinatal asphyxia, based on umbilical cord occlusion for 15 to 18 minutes. These injured *Macaca nemestrina* received rhEPO (Epogen; either EPO 3500 U/kg × one dose i.v. followed by three i.v. doses of 2500 U/kg given at 30 min, 24 h, 48 h and 7 days; or EPO 1000 U/kg/day i.v. × four dosages at 30 min, 24 h, 48 h and 7 days) and they also underwent also hypothermia, that started by the third hour of life and lasted 72 h (33.5 °C). These treated macaques presented a longer survival rate without moderate-severe cerebral palsy, preserved cerebellar growth rates, and exhibited improved neurocognitive behavioral scores after perinatal asphyxia [72]. As the safety and efficacy of multiple EPO doses against HIE have been demonstrated in rodent and primate models, some small human clinical trials have been carried out. Zhu et al. [73] showed how repeated low-dose rhEPO treatment (Roche Diagnostics GmbH) reduces the risk of disability for infants with moderate HIE, improving neurologic outcomes, without apparent side effects. Elmahdy et al. [74] found that the administration of rhEPO (Eprex; 2500 IU/kg, subcutaneously, daily for 5 days) in neonates suffering mild/moderate HIE decreased endogenous NO production, reduced seizures, and alleviated neurodevelopmental disorders to 6 months of age. Similarly, Wu et al. [75] reported that multiple doses of EPO (up to 2500 U/kg, i.v.) given in conjunction with hypothermia are well tolerated in newborns with HIE. Rogers et al. [76] similarly declared that a high dose of EPO in combination with hypothermia does not worsen long-term outcomes, and what is more, this study provided additional confirmation that EPO does not have side effects, at least under these specific conditions. More recently, Wu et al. [77] reported that high doses of EPO given with hypothermia for HIE may result in less brain injury as assessed with magnetic resonance imaging (MRI) and improved motor function in a 1-year follow-up phase II double-blinded, placebo-controlled trial. Therefore, these clinical trials also provide evidence supporting the safety and preliminary efficacy of repeated or high doses of EPO in humans

### 3.3. Resveratrol

Resveratrol (3,5,4′-trihydroxystilbene) is a polyphenol produced by different plant species, such as grapevines, pines and pomegranates, and whose most common dietary source is red wine. This flavonoid is made up of two aromatic rings attached by a methylene bridge. Because of its anti-oxidative, anti-apoptotic and anti-inflammatory effects, resveratrol has been found to be neuroprotective [78,79,80]. The protective effects of resveratrol against brain injury caused by stroke in a middle cerebral artery occlusion model (MCAO) in adult rodents have been investigated in depth and clearly demonstrated [81,82,83,84,85]. The mechanisms of action of this polyphenol in stoke are related to the reduction of oxidative stress by inhibiting xanthine oxidase and preventing the production of hypoxanthine, xanthine and oxygen radicals [83] as well as by increasing the levels of malondialdehyde and reduced glutathione [85]. Likewise, resveratrol also improves brain energy metabolism [83,84] by restoring ATP content and the activity of mitochondrial respiratory complexes [84]. Although research studies in rodent models of neonatal brain injury are not as abundant as in adult stroke models, they strongly support the safety and efficacy of resveratrol for improving histological and functional outcomes after hypoxia–ischemia in neonates, even if the mechanisms of its actions are not completely understood. 

**Figure 3:**
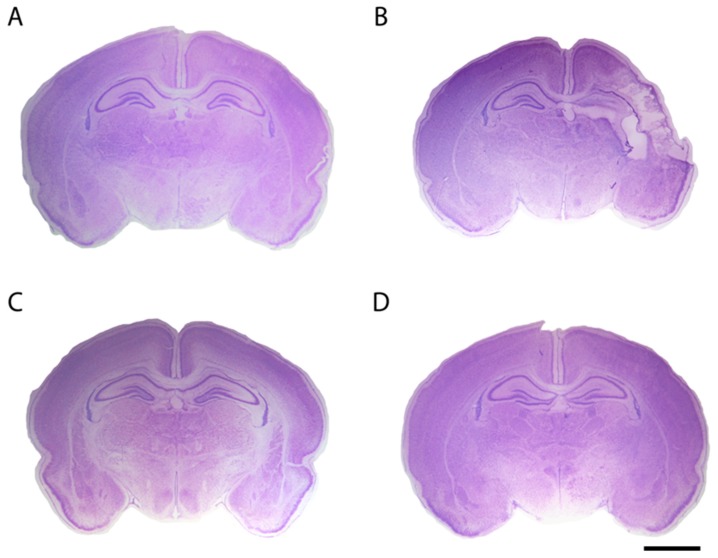
Representative stereomicroscopic photographs of rat brain sections (interaural distance 5.40 mm and bregma −3.60 mm) stained with Nissl at P14 from different experimental groups: (**A**) the control group; (**B**) in the hypoxia–ischemia (HI) group, pups underwent permanent left common carotid artery occlusion and, after two hours of recovery, they were asphyxiated for 135 min in 8% O_2_; (**C**) the HI group pretreated with resveratrol (20 mg/kg), which was intraperitoneally administered 10 min before hypoxia [86]; and (**D**) the HI group pretreated with docosahexaenoic acid (1 mg/kg) injected intraperitoneally 10 min before hypoxia [90]. The brain from the HI group presented an evident loss of tissue in the ipsilateral side, while brains pretreated with either resveratrol or docosahexaenoic acid were similar to the control brain, without obvious histological signs of infarct. Scale bar: 2.5 mm.

Resveratrol, when administered before the HI event, was found to be a potent protective agent that diminished tissue loss (Figure 3) and consequently infarct area both in rats and mice [86,87,88]. It also preserved myelination and minimized the astroglial reactive response [86,87]. Additionally, its neuroprotective effects were found to be long lasting because it was able to improve long-lasting cognitive deficits induced by hypoxia–ischemia [86,87]. Both non-spatial and spatial working memories, assessed by different behavioral tests, were found to be significantly improved in resveratrol-treated neonatal rodents [86,87,88], and these findings might be associated with the preservation of gray and white matter, especially at the level of the cortex and the hippocampus. The beneficial effects of resveratrol to recover the alteration of auditory-evoked potentials and reduce morphological damage in the inferior colliculus after rat perinatal asphyxia have also been reported [89]. Perhaps one of the best known mechanisms of action of resveratrol is that of apoptosis prevention. West et al. [88] reported that caspase-3 activation is minimized by resveratrol. Likewise, it has also been proposed that one of the mechanisms for this neuroprotection may be related to the maintenance of the mitochondrial state, by protecting the integrity of the inner membrane and the transmembrane potential, and by decreasing ROS production [86,89].

In contrast, there are some controversial results regarding resveratrol post-administration efficacy. While Arteaga et al. [86] and West et al. [88] did not observe any signs of neuroprotection with resveratrol administered after hypoxia–ischemia in rodents, Karalis et al. [87] did. However, this discrepancy is likely due to the use of different durations of hypoxia (2 h and 15 min for Arteaga et al., versus 1 h for Karalis et al.) and to the use of different doses of resveratrol (20 mg/kg in the case of Arteaga et al. and West et al., versus 90 mg/kg for Karalis et al.), because of the dose-dependent effects in this animal model [88,91,92]. Hence, Arteaga et al. [86] proposed that low doses of resveratrol post-treatment can alleviate mild HI injury, but not severe or moderate injury. So increasing the concentration or repeating the dose over time could potentially result in neuroprotection against severe HI brain damage.

Regarding the combination of resveratrol and the currently employed clinical treatment of HIE, Toader et al. [93] found that combined therapy had a neuroprotective effect in Wistar rats, significantly influencing oxidative stress parameters, particularly malondialdehyde levels. As resveratrol has been found to protect against stroke in neonatal rats and mice as well as in adults, it could potentially be considered for further investigations in large animal models, focusing on identifying the optimal dose and timing of administration to use it as a post-treatment, which is clinically more relevant.

### 3.4. Omega-3 Fatty Acids

Omega-3 fatty acids are polyunsaturated fatty acids (PUFA) with a double bond (C=C) at the third carbon atom from the end of the carbon chain, hence the name “Ω-3”. As mammals are unable to synthesize Ω-3 fatty acids, they have to obtain them through diet, with marine algae and phytoplankton being their primary sources. The three types of Ω-3 fatty acids involved in human physiology are α-linolenic acid (ALA), eicosapentaenoic acid (EPA) and docosahexaenoic acid (DHA) and each of these has have been found to exert neuroprotective effects against brain injury induced by experimental stroke in adult and neonatal animal models. 

In adult models of ischemia, different Ω-3 fatty acid family members have demonstrated their efficacy. Both oral and intravenous supplementation of ALA improved spatial learning and memory after stroke and this cognitive improvement is correlated with higher survival of hippocampal neurons [94,95,96,97]. Regarding EPA, Ueda et al. [98] established the positive effect of ethyl-EPA on ischemic brain damage following transient focal cerebral ischemia in rats and Okabe et al. [99] demonstrated that EPA prevents memory impairment after ischemia by inhibiting both the inflammatory response and oxidative damage after cerebral ischemia in gerbils.

Likewise, DHA has also shown very optimistic results in this regard, being the most investigated Ω-3 fatty acid. Both pretreatment [100] and post-treatment with DHA [101,102,103,104] are efficient against experimental stroke in adult rats. Similarly, both DHA and its bioactive mediator neuroprotectin D1 improve neurological deficits and reduce infarct size in aged animals [105,106]. Furthermore, DHA complexed to 25% human albumin elicits high-grade neurobehavioral and histological neuroprotection in transient focal and permanent cerebral ischemia in young and aged rats [105,107,108,109].

Concerning neonatal HI brain injury, only Mucci et al. [110] demonstrated how flaxseed consumption, rich in DHA’s precursor α-linolenic acid, during gestation and lactation, was able to mitigate brain mass loss and to improve spatial memory and motor hyperactivity. As far as we know, there is no more evidence about the effects of ALA in neonatal models. Similarly, the neuroprotective effects of EPA when given alone, do not appear to have been investigated, with the exception of its administration in combination with DHA [111,112]. Zhan et al. [111,112] reported that Ω-3 PUFA acid administered as a dietary supplement (DHA + EPA) to pregnant female rats, starting the second day of pregnancy until 14 days after parturition, confers long-term neuroprotection against neonatal hypoxic–ischemic brain injury by attenuating microglia-mediated inflammatory responses through an NF-κB-dependent mechanism [111] and inhibits neuronal cell death by promoting phosphatidylserine formation and Akt signaling [112]. Indeed, both Williams et al. [113] and particularly Mayurasakorn et al. [114] found that triglyceride emulsions of DHA (tri-DHA) but not tri-EPA are neuroprotective when administered immediately after HI injury in neonatal mice.

As in adult models of stroke, DHA has been the most investigated Ω-3, perhaps because it is the major polyunsaturated fatty acid in the adult mammalian brain, where it constitutes more than 30% of the total phospholipid content of cellular membranes. DHA is able to reduce infarcted area under a variety of schedules. When administered as a pretreatment complexed to 25% human albumin (1 mg/kg), besides reducing cerebral damage at the level of the middle hippocampus [90,115] (Figure 3), DHA preserves neurons and myelin and diminishes the astroglial reactive response and microglial activation [90]. DHA is also able to recover the alteration of auditory-evoked potentials and reduce morphological damage in the inferior colliculus after rat perinatal asphyxia [89]. Likewise, positive results were obtained when two doses of n-3 triglycerides (containing both DHA and EPA) [113] and when triglyceride emulsions of DHA were given immediately after HI injury in mice [114]. Post-HI administration of tri-DHA significantly increased DHA content and preserved Ca^2+^ buffering capacity in cerebral mitochondria, ameliorated oxidative brain injury and increased DHA derived bioactive metabolites in brain [114]. On the other hand, Berman et al. [116] reported that when DHA is given 15 min after HI, the treatment improves forepaw placing, but it is not able to reduce brain injury, in spite of the dosage (DHA 1, 2.5, or 5 mg/kg). It has been proposed that DHA for neuroprotection appears to have a reverse dose dependency, with more favorable results being found with low and moderate doses [102,116]. One of the reasons why DHA did not work as a post-treatment might be that multiple DHA doses are required, as in the work of Mayurasakorn et al. [114], where the first dose was administered immediately after HI and the second dose 1 h later. The neuroprotective effects of DHA are long lasting since it ameliorates long-term cognitive impairments related to memory caused by neonatal hypoxia–ischemia in rats [90,111,114,117]. This effect has been observed both when DHA is given prior to and after the injury, and also when it is given as a maternal supplementation starting before the HI injury was induced at P7 and maintaining the Ω-*3* PUFA-enriched diet seven days more after that. One of the proposed mechanisms of action of this neuroprotection may be related to the maintenance of mitochondrial function [89,90,114].

Taken as whole, DHA seems to be a promising candidate to use as an add-on treatment in combination with the standard therapy of hypothermia for reducing the brain damage induced by HI, due to its well-established safety record and its ability to readily cross the blood–brain barrier. In this sense, Berman et al. [118] demonstrated that DHA post-treatment (2.5 mg/kg complexed to 25% albumin) and brief delayed hypothermia (3 h, 30 °C) confers markedly improved sensorimotor function, but only modestly reduced tissue injury in a neonatal rat asphyxia model. Unfortunately, these authors did not clarify either the cellular or molecular mechanisms whereby DHA and hypothermia act in concert to moderately attenuate brain injury and preserve sensorimotor function, and it is not clear if the combination of both confers additive or synergistic neuroprotection. Thus, one of the next challenges in the future should be to determine the appropriate dosage to use in post-treatment in combination with hypothermia and to fully understand the relevant mechanisms of action, since the potential of DHA as an effective therapeutic candidate for reducing ischemia-induced neurodegeneration in the neonate continues to hold promise.

### 3.5. N-Acetyl-l-cysteine

*N*-Acetyl-l-cysteine (NAC) is a well-known and potent thiol-containing antioxidant. It is also a scavenger of oxygen radicals and a precursor of glutathione. NAC is able to scavenge ROS, to restore glutathione levels, to mitigate redox potential, to reduce apoptotic cell death and to diminish both inflammatory cytokines and inducible nitric oxide synthase in an adult rat stroke model [119,120]. Additionally, pretreatment with NAC (i.p., 150 mg/kg given 30 min before) increased HIF-1α protein levels and its target proteins EPO and glucose transporter 3, in the ipsilateral hemispheres of rodents subjected to 90 min MCAO and 24 h reperfusion, indicating that HIF-1α might take part in NAC´s protection in stroke [121].

In the same line, NAC has been demonstrated to attenuate HI brain injury in neonatal rats when administered either alone [122] or in combination with systemic hypothermia [123,124]. When given alone, its administration (100 mg/kg), 30 min prior to carotid artery occlusion surgery at P7 and once a day up to P44, also recovered motor functions and mitigated the demyelination process in the *corpus callosum* of neonatal HIE animals [122]. Moreover, when hypothermia was used in combination with NAC (50 mg/kg) administration by intraperitoneal injection daily until rat sacrifice (at 3 week of age), resulted in improvements in infarct volume, myelin expression and functional outcomes after focal HI injury [124]; moreover, inducible nitric oxide synthase expression and caspase-3 activation in the injured hemisphere were significantly decreased compared to cases treated with hypothermia alone, but only in female rats. When NAC was continued for 6 weeks, starting 1 h after initiation of hypothermia, significant improvement in long-term neuromotor outcomes over hypothermia treatment alone was also observed [123]. The administration of multiple doses of NAC (200 mg/kg) provided marked neuroprotection in a model that combines infection/inflammation and HI. This was associated with the reduction of isoprostane activation and nitrotyrosine formation, with the increase of the levels of the antioxidants glutathione and thioredoxin-2, and with the inhibition of caspase-3, calpain, and caspase-1 activation [125]. NAC has also been shown to exhibit neuroprotection in larger animal models. However, to date, no pilot studies have been carried out in newborn human babies. Post-resuscitation administration of NAC (20 or 100 mg/kg/h) abolished the increased concentration of cerebral hydrogen peroxide and oxidized glutathione levels as well as lipid peroxidation in newborn piglets [126]. Likewise, when it was given in a bolus of 3 mL/kg and followed by a 2 mL/kg/h infusion for 4 h, it significantly reduced cerebral oxidative stress by suppressing hydrogen peroxide but not NO production, improved cerebral perfusion and reduced brain lactate accumulation in moderately hypoxic newborn piglets [127].

### 3.6. Deferoxamine

Deferoxamine (DFO), a bacterial siderophore produced by *Streptomyces pilosus*, chelates free iron thereby preventing hydroxyl formation via the hydrogen peroxide-driven Fenton reaction. DFO has several medical uses; it acts by removing excess iron in the bloodstream and thus by reducing damage to organs [128]. In an in vitro model of ischemia, it has been found that DFO ameliorates neuronal death after oxygen and glucose deprivation, and that this neuroprotection by DFO is, in part, eliminated by blockade of HIF-1, suggesting that DFO protects through iron chelation and HIF-1 induction [129]. As DFO can cross the blood–brain barrier, different research groups have assessed and then demonstrated the effectiveness of this antioxidant against HI brain injury in different animal models. DFO was found to have neuroprotective properties in both postnatal day 7 rats, by decreasing the levels of excitatory amino acids and by improving histological outcome in the hippocampus when administrated immediately after HI (subcutaneous injection of 150 mg/kg) [130], and in P7 mice when administered 10 min and 24 h after the injury (100 mg/kg, subcutaneously) [131]. Accordingly, in newborn lambs, DFO effectively lowered free iron in cortical brain tissue and plasma [132], preserved carotid artery flow, relative cerebral O_2_ metabolism and electrocortical brain activity [133]. Moreover, it maintained cortical cell membrane activity of Na^+^, K^+^-ATPase during early reperfusion after severe HI (2.5 mg/kg, i.v.), pointing to a reduction of free radical formation by DFO [134]. DFO has also been found to be an effective therapeutic agent for reperfusion injury in the brain of newborn piglets after neonatal hypoxia–ischemia (12.5 mg/kg/day) by ameliorating cerebral energy status and the water T2 values [135]. Despite these positive results, the benefits of deferoxamine have never been tested in clinical trials with human infants presenting with HIE.

### 3.7. Melatonin

Melatonin is a naturally occurring hormone that regulates the circadian rhythm. Its neuroprotective effects against neonatal HIE have been investigated in depth due to its potent antioxidant properties [136,137]; it does not have any pro-oxidant effect with other antioxidants nor does it interference with other drug actions [138]. This indolamine acts as a direct scavenger, being able to remove free radicals [139,140], and it can also generate products in a scavenger cascade, which may collectively eliminate up to ten free radicals [140]. Melatonin can also act as an indirect antioxidant through the activation of some of the most important antioxidant enzymes [141], the improvement of mitochondrial efficiency [142], the stimulation of gene expression and through the enhancing of the antioxidant effect of a variety of substances [143]. In addition, this hormone can act by inhibiting adhesion molecules and by reducing polymorphonuclear leukocyte infiltration and inflammatory activation [136].

Melatonin has been shown to exert significant benefits in animal models of HIE. It has the capacity to reduce infarct volume when administered either before or after injury in rats [144,145,146,147] and to increase the number of morphologically well-preserved neurons in the most affected areas of the brain, i.e., in the hippocampus and parietal cortex [144,148,149]. Similarly, it also prevents morphopathological changes at the level of the inferior colliculus, which is correlated with the recovery of the functional changes of the auditory pathway caused by the HI insult [89]. Besides, melatonin is not only able to reduce neuronal damage but also white matter demyelination at the level of the mid-dorsal hippocampus, thalamus and brainstem [89,144,150] and reactive astrogliosis at the level of the mid-dorsal hippocampus, cortex and brainstem [89,144,150,151].

In some of these studies, a repeated dose has shown optimal results [144,145,146,147], and the concentration of 15 mg/kg melatonin starting at 5 min after the HI event and repeated after 24 and 48 h was found to be the most effective. These results suggested that melatonin acts dose-dependently as a neuroprotectant when administered both before and after the ischemic insult [147]. Another important aspect of the neuroprotective effects of melatonin is that they are long-lasting as both brain injury and behavioral outcomes improved even up to adulthood [147].

The potent antioxidant properties of the main secretory product of the pineal gland have been extensively researched in this model. Maternal melatonin administration within 1 h after an ischemic/oxidative episode can prevent ischemia/reperfusion-induced oxidative cerebral damage in neonatal rats [149]. Moreover, it also reduced the levels of desferoxamine-chelatable free iron, isoprostanes, and neuroprostanes, all of which are oxidative stress biomarkers [147,151,152]. Furthermore, treatment with melatonin reduces nitrotyrosine expression in proteins, the formation of which can result in malfunction of these proteins. It also inhibits monocyte infiltration and microglial activation [151]. In an umbilical cord occlusion model in fetal sheep, melatonin has also been found to be able to abolish lipid peroxidation [153], to attenuate the production of 8-isoprostanes, and the activation of microglia [136]. Prevention of increased free radical production after intrauterine asphyxia in fetal sheep has been also shown [136]. These results indicate the important neuroprotective role of melatonin in reducing oxidative damage resulting from HI, and one of the possible mechanisms of action might be its reduction of endoplasmic reticulum stress and the preservation of sirtuin 1 expression in newborn rat neurons after hypoxia–ischemia [154].

Closely related to oxidative stress, mitochondrial impairment is a key feature underlying neonatal HI. Melatonin administration to pregnant rats may prevent ischemia/reperfusion-induced oxidative mitochondrial damage in the premature fetal brain [155]. Indeed, it protects against oxidative mitochondrial damage induced in rat placenta by ischemia and reperfusion as well [156]. However, the group of Berger et al. [157] recently found no improvement of neuronal metabolism in the reperfusion phase with melatonin treatment after HI brain injury in the neonatal rat, suggesting that melatonin treatment may not have protective effects on neuronal metabolism, since this protection has only been reported in astrocytic mitochondrial metabolism.

Taken as a whole, there is convincing evidence that melatonin has beneficial effects against perinatal brain damage in animals. Similarly, novel melatonin derivatives, such a Neu-P11 (a MT1/MT2 receptor agonist), have also been found to be neuroprotective in brain ischemia related models [158]. The neuroprotection obtained by the combination of melatonin with therapeutic hypothermia after transient hypoxia–ischemia in a piglet model of perinatal asphyxia has already been shown [27]. Intravenous melatonin administration (5 mg/kg/h over 6 h beginning 10 min after resuscitation and repeated at 24 h) increases the beneficial properties of cooling by alleviating energy metabolism in the brain (deep grey matter lactate/*N*-acetyl aspartate and lactate/creatine). Indeed, it did not change the heart rate or arterial blood pressure, inotropes, blood electrolytes, or glucose and lactate levels during or after treatment. When melatonin and therapeutic hypothermia are given together, the benefits of the combination were also related to elevated ATP levels in the brain and to the reduction in the number of terminal deoxynucleotidyl transferase dUTP nick end labeling (TUNEL)-positive cells in the thalamus, white matter, internal capsule, putamen and caudate, as well as to the reduction in levels of caspase-3 in the thalamus [27]. 

These results lay the groundwork for future clinical studies in infants; in fact, melatonin’s neuroprotection against perinatal asphyxia has already been demonstrated in a randomized controlled pilot study [159]. The newborns who received five daily enteral doses of melatonin (10  mg/kg) in combination with hypothermia, had fewer seizures and less white matter abnormalities and presented improved survival without neurological or developmental abnormalities at 6 months. Therefore, in the light of the most recent studies, melatonin could represent a potentially safe approach to perinatal brain damage in humans.

### 3.8. Other Antioxidants

A number of other antioxidants have also demonstrated their potential beneficial effects against HIE, but we would like to highlight in particular magnesium sulphate (MgSO_4_) and nicotine. Magnesium sulphate, an inorganic salt, is the main preparation of intravenous magnesium and it has several medical uses. Magnesium minimizes excitotoxic injury by binding to the magnesium site on the *N*-methyl-d-aspartate glutamate channel in vitro [160]. In premature fetal lambs affected by partial occlusion of the umbilical cord, MgSO_4_ preserves the HI-induced diminishment in the levels of the S-100 protein and it did not alter the expression of endothelial tight junction molecules [161]. The authors suggested that MgSO_4_ neuroprotective effects are due to the restoration of blood–brain permeability. In P7 rats that underwent HI and received MgSO_4_, sensorimotor deficits and brain injury were reduced [162]. In contrast, Galinsky et al., [160] in their review about the effectiveness of maternal magnesium sulfate in extensive controlled trials, suggested that magnesium is not consistently neuroprotective for perinatal HI models in term-equivalent preclinical studies.

Nicotine, a nicotinic acetylcholine receptor agonist, is known to have antioxidant functions. When intraperitoneally administered two hours before the HI insult in P7 rats, nicotine (1.2 mg/kg) is able to recover the alteration of auditory-evoked potentials and reduce morphological damage in the inferior colliculus [89], but it scarcely altered the expression of immediate early genes compared to the control group [150]. Although, it has not been deeply investigated in HIE, nicotine may prove to be a promising candidate for further evaluation in the light of the recent results.

## 4. Conclusions

There is convincing evidence in the above mentioned literature that different antioxidant treatments have the potential to be therapeutically effective in different animal models of HIE. Nevertheless, we would like to point out that none of the mentioned antioxidants reviewed have been approved either by the Unites States Food and Drug Administration or by the European Medicine Agency. Most of the revised antioxidants do not present negative side effects at high doses and almost all of them seem to be neuroprotective both when used as pre-treatments or post-treatments. However, for some of them, multiple doses are required to achieve optimal protective effects. Although the mechanisms by which antioxidants exert neuroprotection are not fully understood, in part also because of the complexity of neonatal hypoxic–ischemic pathophysiology, these studies have demonstrated the high potential of these antioxidants as efficient therapeutic agents for the reduction of brain damage in newborns. The most promising candidates seem to be the antioxidants that have already been found to be neuroprotective in human trials, i.e., the erythropoietin and melatonin, since their beneficial effects against HIE have been proved when they are used as an add-on treatment in combination with hypothermia. Additionally, these antioxidants exhibit anti-inflammatory and anti-apoptotic properties as well. The next challenging goal will be to adjust the optimal dosage of these antioxidants when given in combination with hypothermia, so that larger neuroprotective effects can be exerted in the human brain. To this end, additional randomized controlled human trials will need to be performed.
